# Microbes: Food for the Future

**DOI:** 10.3390/foods10050971

**Published:** 2021-04-28

**Authors:** Matilde Ciani, Antonio Lippolis, Federico Fava, Liliana Rodolfi, Alberto Niccolai, Mario R. Tredici

**Affiliations:** Department of Agriculture, Food, Environment and Forestry (DAGRI), University of Florence, Piazzale delle Cascine 18, 50144 Florence, Italy; matilde.ciani@unifi.it (M.C.); antonio.lippolis@stud.unifi.it (A.L.); federico.fava@stud.unifi.it (F.F.); liliana.rodolfi@unifi.it (L.R.); alberto.niccolai@unifi.it (A.N.)

**Keywords:** microbial protein, HOB, methanotrophs, mycoprotein, yeasts, cyanobacteria, microalgae cultivation with artificial light

## Abstract

Current projections estimate that in 2050 about 10 billion people will inhabit the earth and food production will need to increase by more than 60%. Food security will therefore represent a matter of global concern not easily tackled with current agriculture practices and curbed by the increasing scarcity of natural resources and climate change. Disrupting technologies are urgently needed to improve the efficiency of the food production system and to reduce the negative externalities of agriculture (soil erosion, desertification, air pollution, water and soil contamination, biodiversity loss, etc.). Among the most innovative technologies, the production of microbial protein (MP) in controlled and intensive systems called “bioreactors” is receiving increasing attention from research and industry. MP has low arable land requirements, does not directly compete with crop-based food commodities, and uses fertilizers with an almost 100% efficiency. This review considers the potential and limitations of four MP sources currently tested at pilot level or sold as food or feed ingredients: hydrogen oxidizing bacteria (HOB), methanotrophs, fungi, and microalgae (cyanobacteria). The environmental impacts (energy, land, water use, and GHG emissions) of these MP sources are compared with those of plant, animal, insect, and cultured meat-based proteins. Prices are reported to address whether MP may compete with traditional protein sources. Microalgae cultivation under artificial light is discussed as a strategy to ensure independence from weather conditions, continuous operation over the year, as well as high-quality biomass. The main challenges to the spreading of MP use are discussed.

## 1. Introduction

As concerns future food demand and supply, most analysts agree on the necessity to increase food production by more than 60% in the next three decades to satisfy the needs of the world’s growing population and income-dependent global dietary shift (e.g., increased consumption of animal protein) [1,2]. Most studies highlight the challenges of achieving such an increase and moving, at the same time, towards a sustainable global food system in the face of urbanization, scarcity of natural resources, and climate change [2,3].

The food system is indeed a major driver of biodiversity loss, land-use change, depletion of freshwater resources, and pollution of aquatic and terrestrial ecosystems, mainly through nitrogen and phosphorus run-off from synthetic fertilizers and manure applications [4]. Planetary boundaries define the thresholds below which humanity should stay in order to maintain planetary life support systems in a safe operating space, thus avoiding triggering a catastrophic cascade of dreadful changes in natural earth systems [5]. Agriculture significantly contributes to the crossing of three of these boundaries: biodiversity loss and biogeochemical flows of nitrogen and phosphorus. The current food chain ensures globally a net excess of protein (of more than 80%) and calories (of about 8%), yet more than 800 million people are afflicted by food scarcity, more than 2 billion suffer from malnutrition (lack of protein, vitamins and minerals, obesity), and the environmental costs of food production are no longer sustainable [6,7]. 

According to many projections, an increase in food production of 50% with current agricultural technologies will result in an 80% increase in greenhouse gas (GHG) emissions [7,8]. In particular, reducing the consumption of animal products will be a key determinant to avoid further biodiversity losses, limit adverse impacts of agriculture on soil, water and atmosphere, and mitigate climate change as the livestock sector appropriates 80% of agricultural land (4.0 out of 5.1 billion hectares) and accounts for 29–43% of the total agricultural water footprint, 46–76% of agricultural GHG emissions, and 34–58% of total nitrogen use [9]. Despite this huge footprint, the livestock sector returns as edible food only a minor share of the protein and calories it consumes [7,10]. 

We need to raise public awareness of the link between food choices and environmental sustainability of current agricultural production patterns and “rethink the whole agri-food system” [7]. Innovative technologies are urgently needed to improve the efficiency of the food production system and reduce the negative externalities of agriculture and, in particular, of meat production. The replacement of animal-based products, at least partially, with plant and novel protein sources, e.g., microbial protein (MP) cannot be postponed. The use of microorganisms for fermented foods dates back to our first ancestors, but the use of microorganisms as food sources has received little attention until recently. Only in the last years, MP has gained interest as ingredient for food products (e.g., meat replacers). The pivotal role that microorganisms could play in the transition towards a more sustainable food production system prompted us to review the current state of their use as alternatives to conventional sources of food and feed. The potential benefits and challenges of MP have been analyzed by comparing the environmental impacts and costs of four microbial-based products with other protein sources. Emphasis has been given to the concept of “microbial urban farming’’ and to the possibility of up-cycling recovered carbon and nutrients into MP, as well as to cultivating microalgae under artificial light.

## 2. Microbial Protein (MP)

The term “microbial protein” (MP) refers to microbial biomass used as a source of food or feed. MP has, in general, a high protein content (up to 75% on dry biomass), contains all the essential amino acids, and is rich in vitamins and minerals and various other nutritionally valuable substances [11]. MP can be produced under optimal conditions in closed and intensive systems called “bioreactors”, which differ in construction and functioning according to whether the organism is a phototroph or a chemotroph, an autotroph or a heterotroph. Producing biomass in a bioreactor is much more efficient than cultivating plants in an open field or raising animals, owing to the stability of growth parameters, efficient utilization of nutrients (which can be supplied to exactly match demand), low water and land footprint, and no need for pesticides or antibiotics [1,12,13]. One of the major environmental benefits associated with food production from microbial biomass lies in the efficient use of nitrogen, phosphorus, and other nutrients. Conventional agriculture-based protein production converts only a fraction of the supplied nitrogen into plant and animal protein [1]; the remaining nitrogen is lost to the environment causing contamination of aquifers, eutrophication of surface waters, ocean acidification, and GHG emissions [14]. In bioreactor-based MP production, almost all of the supplied nitrogen (and of the other nutrients) ends up as consumable protein with minimal impacts on the environment. MP production does not directly compete with crop-based food commodities for fertile soil and freshwater and can be located in marginal lands and in industrial or metropolitan areas. 

In about thirty years from now, urban areas will host more than two-thirds of the world population [2]. Feeding millions of people living amassed in a restricted urban location and disposing their wastes will be some of the main challenges of the next decades. Megacities and industrial areas pose the necessity and offer the opportunity, by means of MP production and vertical farming, to recover most of the nutrients and energy embedded in solid urban wastes or leaving the urban area via the sewage system to be further recycled into sustainable protein sources.

**Figure 1:**
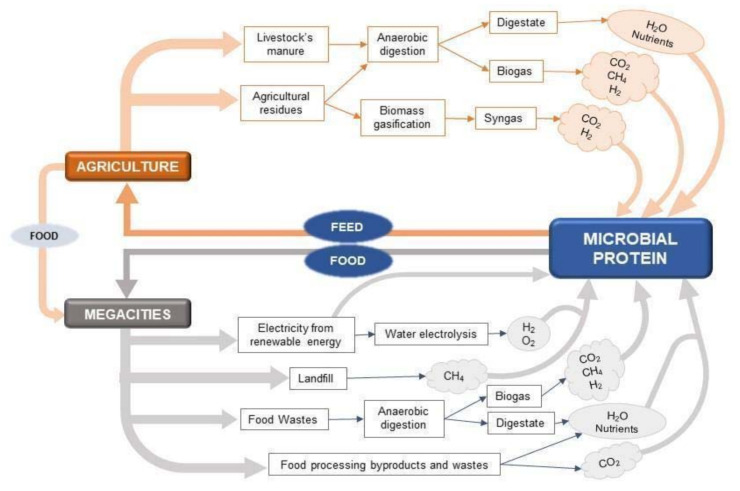
Fluxes of nutrients, water, electric and chemical energy, organic and inorganic carbon from agriculture (above) and megacities (below) wastes to microbial protein production.

The implementation of “urban microbial farming” does not necessarily rely on the direct use of fossil fuels and offers an interesting alternative to the traditional Carbon Capture and Utilization (CCU) technologies, since autotrophic microorganisms need an inorganic carbon source (CO_2_) to grow and produce biomass. Thus, some MP can be part of strategies that exploit carbon dioxide emitted from industrial point sources (e.g., flue gas from power stations, incinerators, cement factories, emissions from food processing, anaerobic digestion of plants, etc.) [13,15,16]. MP, as other conventional protein sources, can be produced without resorting to fossil fuels, but using solely renewable energy such as photovoltaic- or wind-based electricity. Wind turbines and photovoltaic panels, located in megacities, industrial areas, marginal lands, or offshore, can provide the needed electricity for bioreactor operation, biomass harvesting, and processing. Electricity can be used for H_2_ production (an electron source for some chemo-lithotrophic bacteria) or converted into PAR light (radiation with wavelength from 400 to 700–1100 nm) to grow phototrophic microorganisms in photobioreactors [12]. Moreover, MP offers solutions for recovering carbon, energy, and nutrients from crop and food processing residues or animal sludge and manure. Anaerobic digestion or biomass gasification can convert such substrates in biogas or syngas, yielding gases such as CO_2_, H_2_, and CH_4_ exploitable by microorganisms as carbon and/or energy sources. The mentioned options are represented in Figure 1. The scheme proposes a combination of processes in which agricultural and urban wastes are used as nutrients, energy, and carbon sources for MP production in a fully integrated circular bio-economy approach.

## 3. MP from H_2_-Oxidizing Bacteria (HOB)

The use of HOB for food is amongst the most challenging, yet promising, technologies of the future bio-economy [11,17]. HOB are mostly chemo-litho-autotrophs, i.e., they use inorganic electron (H_2_) and carbon (CO_2_) sources to grow and produce biomass, offering large CO_2_-emitting industries a tool to reduce their carbon footprint and produce, at the same time, feed, food, or green chemicals [17]. One key factor that characterizes MP from HOB is that their production can be decoupled from fossil fuels, being possible to exploit solely renewable energy for H_2_ and O_2_ production, both needed by HOB for cellular energy generation [15]. H_2_ and O_2_ can be produced from water electrolysis by electricity obtained from solar or wind-based installations at costs that are rapidly decreasing, or from biomethane reforming [15]. HOB-based protein produced in high-density reactors presents environmental footprints several orders of magnitude lower than plant- and animal-based protein production (Table 1). Being able to achieve a conversion of 2.4 kg of dry biomass per kg of H_2_, HOB can yield hundreds of tonnes of biomass per hectare per year [18]. The high protein content (up to 75% on dry weight basis) of HOB biomass is coupled with an amino-acid profile closer to high-quality animal rather than to vegetable protein, and with high protein assimilation in the gastro-intestinal tract (1.4 higher than wheat proteins and almost comparable to casein) [18]. The production cost of HOB biomass has been estimated at EUR 2.5–5 kg^−^^1^ [16], which compares favorably with that of beef and fish (Table 1). Considering that more than 60% of the total cost is due to hydrogen, it appears likely that the technical advances in the energy sector and in water electrolysis (e.g., more efficient new materials and electrolyzers) will significantly reduce HOB costs in the future [16,18] making this MP source reach economic feasibility. Solar Foods (Laskunet, Finland) has announced that they will bring to market a food product from HOB by 2021 [17].

## 4. MP from Methanotrophic Bacteria

Methanotrophic bacteria (methanotrophs) use methane (CH_4_) as an energy and carbon source. Unlike HOB production, still in its infancy, the production of MP from methane was already achieved at industrial scale in the 1970s when MP was successfully tested as protein-rich feed additive for ruminants, pigs, and chickens [11]. The advantage of using methane for MP production is that it can be attained, together with CO_2_, from biomasses or waste streams (e.g., urban organic wastes, sewage sludge, food processing wastes, agricultural residues) through anaerobic digestion [17]. The main driver leading to the successful application of methane-based protein has been the aquaculture sector. In 2014 Unibio (England, UK) has launched the EFPro (Environmentally Friendly Protein Production) project aimed to produce an MP (called Uniprotein^®^) from *Methylococcus capsulatus*. The product contains more than 70% protein with all the essential amino acids. Another company, Calysta Inc. (Teesside, England, UK), produces several tonnes of MP from *M. capsulatus* marketed under the name of FeedKind^®^. The final product is comparable to fishmeal in terms of nutritional value and essential amino acid content [11]. FeedKind^®^ shows great advantages in terms of sustainability when compared to animal or plant-based feed. The negligible land footprint and a water footprint about two times lower than that of fishmeal and 14 times lower than that of soybean meal (Table 1) highlight the advantages of producing feed from methanotrophic bacteria in terms of environmental sustainability [42]. Calysta Inc. uses an innovative fermentation process, which allows productivities of 3–4 kg (dry matter) of MP per m^3^ of reactor volume per hour [11]. The production cost of Feedkind^®^ was reported to be about EUR 1.5 kg^−^^1^, which compares favorably with that of fishmeal, beef and insects (Table 1).

## 5. Fungal MP

The production of MP from fungi is a commercial reality. Among microorganisms, yeasts are probably the first domesticated group in the history of humankind. Yeasts have been used for the production of fermented beverages, such as beer and wine, and of bread, which archaeologists date to thousands of years ago [43,44]. The long-time use of yeasts in food and beverages has favored a higher acceptance of fungi as food ingredients compared to other microorganisms [45]. The potential use of yeasts as a feeding supplement was investigated for the first time at the beginning of the 1900s in Germany. During the following years, different processes to increase biomass yield were explored and a variety of yeasts were adopted as food and feed supplements [46]. During World War II, yeasts were incorporated into army, and then into civilian, diets. By 1960, 250,000 tonnes of yeast were being produced in different countries of the world as support to agricultural protein supply [46]. Yeast extracts produced from brewer’s spent grain (e.g., Marmite^®^ by Unilever PLC (London, UK) and Vegemite^®^ by Bega Cheese Ltd. (New South Wales, Australia) have been sold for more than a century [35]. Marmite^®^ is a creamy product, containing 34% protein and B vitamins, sold at a retail price of about EUR 12 kg^−^^1^ [47], corresponding to EUR 34 kg^−^^1^ protein. Yeasts are also sold as dietary supplements, flavoring agents, and feed [35]. One million tonnes of yeast are produced annually in the European Union [48]. The production of 1 kg of dry yeast consumes 42 MJ [49] and generates 3.24 Kg CO_2_-eq. [48]. Recent studies have investigated the use as feedstock for yeast cultivation of different agro-industrial wastes (e.g., sugarcane bagasse, hemicellulosic hydrolysate, whey, orange, and potato residues) to increase the environmental sustainability of yeast production [35,48]. *Saccharomyces cerevisiae* is the species mainly used by industry, although other yeasts (e.g., *Candida tropicalis, Candida utilis*) are also cultivated for MP since their biomass is particularly rich in protein and vitamins [35,45].

The most notable rediscovery of MP after the 1980s regards a mycoprotein which has been on sale in the United Kingdom since 1985 and is now marketed in most of the European countries under the brand name of Quorn™ (Monde Nissin Corporation, Philippines), mainly in the form of burgers, slices, and nuggets [33,46]. Quorn™ is made starting from a culture of the aerobic ascomycete *Fusarium venenatum*. The fungus is cultivated under sterile conditions in large fermenters to obtain a paste termed mycoprotein (about 50% protein in dry weight). The culture is processed through a thermo-physical treatment to reduce its nucleic acid content, and the mycelium is separated by centrifugation from the growth medium. After the addition of ingredients to give a suitable texture and improve the taste, it is used in the manufacturing of food products [33]. In contrast to HOB, fungi need an organic carbon source (e.g., sugars) for growth. Hence their cultivation cannot be decoupled from freshwater and arable land use. The culture medium of *F. venenatum* for Quorn™ production contains glucose syrup from wheat, but the production of fungal protein could also use organic substrates recovered from agricultural residues or food-wastes in a prospective of energy and nutrient recycling. Mycoprotein production shows much higher sustainability than meat. A cradle-to-gate life cycle analysis (LCA) carried out by Carbon Trust for Quorn™ mince estimated carbon, water, and land footprints at least ten times lower than those for beef and four, three, and two times lower than those for chicken, respectively [33]. Mycoprotein is a source of high-quality protein [17] (all the essential amino acids are present) with a biological value similar to that of milk protein, coupled with a low energy and high fiber content. Typically, 100 g of dry mycoprotein contains 45 g protein, 25 g fiber, 13 g fat, and 10 g carbohydrate, plus a range of vitamins of the B complex and minerals such as calcium, phosphorus, magnesium, iron, and zinc [33]. It has been reported that consumption of MP from *F. venenatum* causes no health concerns [33]. Currently, Quorn™ production amounts to about 25,000 tonnes (dry mass) per year with global retail sales of about EUR 200 million [11]. The market value of mycoprotein is expected to increase by 20% annually in the coming years [11].

## 6. MP from Microalgae

Microalgae (including cyanobacteria) are photoautotrophic microorganisms, which use CO_2_ as carbon source and perform oxygenic photosynthesis to obtain energy from sunlight or artificial light. Cyanobacteria, in particular the *Arthrospira* and *Nostoc* species, have been used in Africa and Asia as food for centuries [50,51]. The interest in microalgae as food is due to their high nutritional value [52]. They can have high digestible protein contents with a balanced amino acid composition [53,54], good content of vitamins, minerals, carotenoids, and polyunsaturated fatty acids [55,56]. Furthermore, microalgae show many bioactivities including anti-inflammatory, antibacterial, antioxidative, hypolipidemic, and anti-carcinogenic activity, which make them attractive in the nutraceutical and pharmaceutical fields [57,58,59,60].

Commercial microalgae production started about sixty years ago with *Chlorella* in Japan. Today, *Chlorella* (mainly *C. vulgaris*, *C. sorokinina,* and *C. pyrenoidosa*) with about 5000 tonnes year^−1^ and spirulina (various *Arthrospira* species) with about 15,000 tonnes year^−1^ comprise over 80% of the world microalgal biomass production, which, without considering phytoplankton produced in hatcheries, totals about 25,000 tonnes annually [61]. The other commercially cultivated microalgae are *Dunaliella* (about 2000 tonnes year^−1^) and *Haematococcus* (about 1000 tonnes year^−1^). These microalgae (and derived products) are mostly sold for human food, nutraceuticals, and feed ingredients. In China, 200 tonnes (fresh weight) of the cyanobacterium *Nostoc sphaeroides* are also annually produced and sold as a traditional food and for use in cosmetics [61]. Another cyanobacterium, *Aphanizomenon flos-aquae*, is commercialized worldwide (about 500 tonnes annually), although it is not industrially cultivated and its biomass is harvested from lake Klamath (OR, USA) [62]. Only few microalgae, namely, *Arthrospira platensis*, *A. flos-aquae*, *Chlorella luteoviridis*, *C. pyrenoidosa,* and *C. vulgaris*, diffusely used prior to May 1997, are authorized as food in Europe [63]. In 2005, the diatom *Odontella aurita* was also approved as food [64]. Recently, *Tetraselmis chuii* and astaxanthin from *Haematococcus pluvialis* were also authorized [65,66]. These microalgae can be found in the market in the form of tablets, powders, capsules, and pills, or incorporated into food products (e.g., pastas, bakery products, snack foods, candies, yoghurts, soft drinks) [67,68,69,70,71,72]. Different companies are currently investing in this innovative microalgae-based food/beverage sector, such as Terravia Holdings, Inc. (ex-Solazyme, San Francisco, CA, USA) and Vicky Foods (Valencia, Spain, ex-Dulcesol Group,), and are developing baked products with *Chlorella* [73]. The French company Roquette Frères commercializes “Algility™ Chlorella” composed of dried *C. sorokiniana* biomass. Among the world’s largest producers of *Arthrospira* there are Earthrise^®^ Nutritionals (USA), Cyanotech Corporation (USA), Hainan Simai Pharmacy (China), Yunnan Green A Biological Project (China), Inner Mongolia Rejuve Biotech (China), Far East Bio-Tec (China), Qingdao Haizhijiao Biotechnology (China), DIC Corporation (Japan), Parry Nutraceuticals (India), Algene Biotech (India), and Australian Spirulina (Australia) [61,74]. The largest producers of *Chlorella* biomass are Taiwan Chlorella Manufacturing (China), Vedan Biotechnology (China), Far East Bio-Tec (China), Parry Nutraceuticals (India), Daesang Europe (The Netherlands), Corbion (The Netherlands), and Roquette Klötze (Germany) [61]. 

The global microalgae market is estimated at about USD 800 million annually [61] and is increasing. *Arthrospira* and *Chlorella* bulk selling prices range from about USD 10,000 to USD 30,000 per tonne (EUR 8.5–25.5 kg^−^^1^) [75]. *Dunaliella salina* and *H. pluvialis* are sold at much higher prices (>USD 100,000 per tonne) for their high content of high-value pigments. Production costs of spirulina (*Arthrospira)*, estimated for a commercial-scale plant in the US, are on average USD 10,000 per tonne plant-gate [75]. The costs rise to EUR 40–50 kg^−^^1^ when the algae are produced in photobioreactors [13,37]. Considering that soybeans sell at USD 300–400 per tonne, even for the “microalgal specialties”, rich in polyunsaturated long-chain fatty acids or carotenoids, it will be difficult to enter in the food/feed market without a significant reduction in production costs. On the other hand, it should be emphasized that aquaculture needs more than 1,000,000 tonnes year^−1^ of phytoplankton for shrimp, fish-larvae, shellfish, and zooplankton rearing. However, at present microalgae biomass commercialization still remains confined to niche-markets with limited applications in feed integration (e.g., pet foods). 

Some *Chlorella* species are able to grow heterotrophically in darkness using organic carbon sources. Under these conditions, cell concentrations in the range of 150–200 g dry biomass L^−^^1^, by far higher than when *Chlorella* is grown photoautotrophically (5–10 g L^−^^1^ as maximum), can be attained. In some commercial plants, cultivation in darkness precedes cultivation in the light; the first phase is carried out to accelerate inoculum production needed to inoculate the photobioreactors in which mass cultivation occurs. Heterotrophic production of *Chlorella* is estimated to be around 500 tonnes per year compared to the 4000–5000 tonnes produced phototrophically. 

### Microalgae Cultivation with Artificial Light

Commercial production of microalgae and cyanobacteria is generally carried out in open ponds under natural light. Inocula and small amounts of cultures for specific uses (e.g., pharmaceuticals, labelled molecules) are produced with artificial light. It is difficult to envisage a process of artificial light conversion into algal biomass able to compete with photosynthesis under sunlight in terms of efficiency and cost. With artificial light, instead of one single step to convert solar photosynthetically active radiation (PAR) into biomass, we need three subsequent steps, each one with its own efficiency. For example, by adopting photovoltaic panels (PV) for electrical energy generation, we need a first step of solar light conversion to electricity, a second step to convert electricity to PAR photons, and a final step in which PAR photons are converted by photosynthesis into the chemical energy of biomass. Outdoors under sunlight, the theoretical photosynthetic efficiency (PE) on total solar radiation is about 12%, but the best average PE values in commercial plants rarely exceed 1.5% [12]. In one of the sunniest locations in Italy, Southern Sicily at 37° N latitude, with an average solar radiation of 66,300 GJ ha^−1^ year^−1^, this efficiency will allow storing into algae biomass about 1000 GJ ha^−1^ year^−1^, equivalent to 50 tonnes per hectare per year of algal biomass with a typical energy content of 20 MJ kg^−1^. Since we have to consider that the land occupied by the microalgae plant is not entirely devoted to cultivation, but some space (about 20%) is needed for roads and ancillary equipment, the yield will be reduced to about 40 tonnes per hectare per year, of which at least half can be protein. Still a considerable outcome compared to crops.

**Figure 2:**
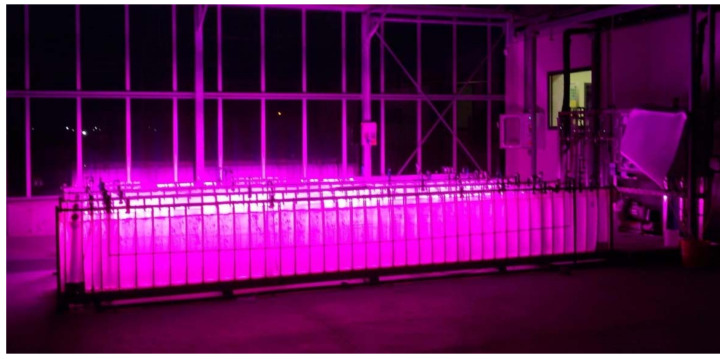
Green Wall Panels^®^ (Fotosintetica & Microbiologica S.r.l.—Florence, Italy) illuminated by LED (Courtesy of Azienda Agricola Serenissima—Padova, Italy).

Under artificial light much higher light conversion efficiencies can be achieved because a homogeneous and relatively low photosynthetic photon flux density (PPFD) can be supplied. In theory, if all supplied photons are PAR, a PE of 27% can be reached [12]. At a PPFD of 300 µmol photons m^−2^ s^−1^ (about 1/6 of solar irradiance during the central daylight hours), the yield of about 1 g biomass per mole PAR photons is attainable [76], which means a PE of about 9.7% (The energy content of 1 g of biomass is 20 kJ; the energy content of 1 mole PAR light (violet LED) is about 206 kJ. PE is 20/206 = 9.7%). At this efficiency, to produce 40 tonnes of biomass we need 40 × 10^6^ moles PAR photons. Light emitting diodes (LED) with an output of 2.7 µmol PAR photons W^−1^ are available today, which can deliver 9.72 moles PAR photons per kWh of electricity consumed ( In energy terms, it is equivalent to a conversion efficiency of electrical energy into light energy of about 56% since 9.72 moles of PAR photons at 206 kJ mol^−1^ (violet LED) = 2 MJ (0.56 kWh)) (Figure 2). Hence to obtain 40 × 10^6^ moles of PAR photons we need 4,115,226 kWh (or 14,814,814 MJ) of electrical energy. With the latest high-efficiency PV panels (e.g., SunPower panels with 19.7% efficiency, net of system energy losses) 75,202,101 MJ of solar energy are required to obtain this electric output, which, at 37° N latitude, can be harvested from one hectare of land area. 

The conclusion is surprising: an area covered with PV panels that convert the impinging solar radiation into electricity, which is subsequently used by LED to attain PAR photons and then biomass by means of indoor bioreactors, can produce a quantity of algal biomass almost equal to that attainable from open ponds or bioreactors deployed under direct sunlight in the same surface area.

Instead of the single step with 1.5% efficiency that characterizes outdoor production with natural light, using artificial light requires three subsequent steps (of efficiency 22.7%, 56%, and 9.7%), whose overall efficiency equals 1.23%. It seems, at a first glance, that there is no advantage in using artificial light, but we need to consider all the benefits of not relying on sun as the energy source, among which are the independence of weather conditions and continuous operation over the year, constant production and reliability of supply, high and constant biomass quality, lower costs for thermoregulation, the possibility to adjust biomass composition and enhance the content of target products, the possibility to satisfy algal species requirements in terms of light quality and intensity, and expected better performances as PV and LED efficiencies improve and costs decrease. Moreover, electricity could be derived from other renewable sources such as hydroelectric plants or wind farms, which have a lower land footprint and lower costs than PV [77]. 

Using the above numbers and with electricity obtained at a cost of EUR 0.045 per kWh, it results that the expenses for light generation (4,115,226 kWh) to produce 40 tonnes of biomass amount to EUR 185,185 and impact on the biomass production cost for EUR 4.6 kg^−1^. This makes artificial light an option only when high-value algal products are the target. Advances in the field of PV have been huge in the last years and electricity at a cost of EUR 0.01 kWh^−1^ seems attainable [78]. If this goal is achieved, the contribution of artificial light to the cost of algal biomass production will decrease to about EUR 1 kg^−1^ making this approach economically competitive even for commodities (e.g., food).

## 7. Productivity, Use of Resources and Impacts of Different Protein Sources

Protein productivity and the impacts of different protein sources (animals, plants, and MP) are shown in Table 1. Bacteria (spirulina, HOB and methanotrophs) show the highest protein content (46–75%). It is worth mentioning that some novel protein sources (e.g., bacteria and insects) present an average protein content which is 2–2.5 times that of meat and 1.7 times that of soybeans. In terms of land protein productivity (kg protein ha^−1^ year^−1^), bacteria attain yields about 600 times higher than those of animals and about 90 times higher than that of soybeans. The use of energy (MJ kg protein^−1^) for meat (beef, pork, and poultry) and insect production is more than five times that for spirulina, whereas mycoprotein-based products show similar values. Soybean proteins require significantly lower energy inputs. As concerns water use (L kg protein^−1^), the lowest requirements are those for bacterial MP, in particular those for methanotrophs that need 3000 times less water than animals. Soybeans, mycoprotein, and cultured meat show intermediate requirements. The major GHG emitter is by far beef (with an average of 440 kg CO_2_-eq kg protein^−1^) followed by pork and poultry (about 40 kg CO_2_-eq kg protein^−1^). Very low emissions characterize soybeans, with an average of 0.6 kg CO_2_-eq kg protein^−1^, values comparable to those of HOB. Although the market of MP is relatively new, the price of protein from microbial sources well compares with that from animal sources, with prices of proteins from methanotrophs being the lowest (EUR 3 per kg of protein). More efforts are needed to meet the prices of soybean protein.

## 8. Conclusions

Although the substitution of meat with MP would strongly reduce the use of arable land, consumption of freshwater, use of antibiotics, pesticides and fertilizers, biodiversity loss, and GHG emissions, there are still major barriers that hinder shifting away from a meat-based diet. MP has still to gain public acceptance and become competitive on the market. One of the main challenges will be to transform microbial biomass into a food that, besides nutritional qualities, has pleasant taste and flavor, and is competitive in terms of cost with animal derived protein (milk, eggs, cheese, and meat). At present, from an economic point of view, the use of MP in feed for livestock is justified only in some market niches, such as aquaculture. Finally, before entering the market, novel MP needs to be authorized by the European Food Safety Authority (EFSA). The spreading of the MP market will mostly depend on a favorable legislation, public acceptance, and low costs, but considering the externalized environmental impacts of the current agri-food production system, the use of microbes as food/feed is worth further exploration.

## Figures and Tables

**Table 1:** Protein content, productivity, use of resources, GHG emissions of traditional and innovative protein sources.

	Protein Content (%) ^a^	Land Productivity ^b^ (kg prot ha^−1^ y^−1^)	Use of Resources			GHG (kg CO_2_-eq kg prot^−1^)	Bulk Price (EUR Kg^−1^)	Price per kg of Protein (EUR kg prot^-−1^)
			Land Use (m^2^ kg prot^−1^)	Energy Use (MJ kg prot^−1^)	Water Use (L kg prot^−1^)			
**Animal proteins**								
Beef	25 [15]	2.4–132 10–20 [19]	76–166 [20] 200–4160 [21]	26.9–210 [21] 179–283 [20]	112,000 [22]	48–839 [21]	4.04 [23]	16.16
Pork	20 [15]	29–250 70–80 [19]	40–76 [20] 69–341 [21]	77.5–329 [21]	57,000 [22]	18.5–64.2 [21]	1.06 [23]	5.30
Poultry	31 [15]	75–333	30–134 [21]	83.3–247 [21]	34,000 [22]	12–60 [21]	1.54 [23]	4.97
Fish	17–20 [24]	322–833 70–210 [19]	12–31 [20]	67–630 [20]	-	4–173 [21]	3.70–28 [25]	18.50–164.70
Eggs	13 [24]	74–385	26–135 [20,21]	10.4–210 [21]	29,000 [22]	11–56 [21]	1.38 [26]	10.61
Milk	3.5 [24]	91–1000 60–70 [19]	1–110 [21]	17.5–383 [21]	31,000 [27]	13–73 [21]	0.35 [28]	10.0
Insects (mealworms or superworms)	45–53 [29]	588–714 180–420 [19]	14–17 [29]	156–186 [29]	23,000 [27]	13–17 [29]	3.70 [30]	6.98–8.22
Cultured meat	19 [31]	435–10,000 70–350 [19]	0–23 [20]	164–555 [20]	1734–4420 [31]	10–40 [20]	54–91 [32]	284.21–478.94
**Vegetable proteins**								
Soybeans	35 [15]	909–1124	8.9–11 [21]	2.1–20.5 [21]	6034–7428 [22]	0.2–1 [21]	0.33 [23]	0.94
Wheat	12 [15]	38–1492	6.7–260 [21]	5.2–51.4 [21]	15,042–15,567 [22]	0.9–20.6 [21]	0.20 [23]	1.67
**Microbial proteins**								
Mycoprotein (fresh)	11.25 [33]	662.25	15.1 [33]	-	6000 [33]	14.2 [33]	-	-
Quorn^TM^ mince and pieces ^c^	13.8–14.5 [33]	370–454 1644–1834	22–27 [33] 5.45–6.08 [34]	147–154 [34]	12,000–14,000 [33]	25–40 [33] 38.27–44.56 [34]	5.86 ^d^	36–38
*Arthrospira platensis* (spirulina)	46–65 [17,35]	5000–18,181	0.55–2 [20]	15–45 [13,20]	800–3000 [13]	1–5 [13,20,36]	5 [37]	7.7
HOB Solein^®^	65–75 [38]	60,000 [38]	0.17	-	200 [38]	0.4 [38]	5 [39]	7.6
Metanotrophs Feedkind^®^	71 [40]	192,307	0–0.052 [40]	-	10–29 [40]	2.23–2.65 [40]	1.52 [40]	3

^a^ The percentage of protein is referred to dry or fresh weight depending on the way in which the product is available on the market. ^b^ Land productivity was calculated from land use values or as reported by [19]. ^c^ Quorn™ products refers to mince and pieces with a content of mycoprotein of about 95%. ^d^ Quorn™ retail price [41].

## Data Availability

The data presented in this study regarding the use of artificial light are available on request from the corresponding author.
